# The Efficacy of Carvedilol in Comparison to Propranolol in Reducing the Hepatic Venous Pressure Gradient and Decreasing the Risk of Variceal Bleeding in Adult Cirrhotic Patients: A Systematic Review

**DOI:** 10.7759/cureus.43253

**Published:** 2023-08-10

**Authors:** Lana Dardari, Maher Taha, Purva Dahat, Stacy Toriola, Travis Satnarine, Zareen Zohara, Ademiniyi Adelekun, Kofi D Seffah, Korlos Salib, Ana P Arcia Franchini

**Affiliations:** 1 Internal Medicine, California Institute of Behavioral Neurosciences & Psychology, Fairfield, USA; 2 Medical School, St. Martinus University, Willemstad, CUW; 3 Pathology, California Institute of Behavioral Neurosciences & Psychology, Fairfield, USA; 4 Pediatrics, California Institute of Behavioral Neurosciences & Psychology, Fairfield, USA; 5 Family Medicine, California Institute of Behavioral Neurosciences & Psychology, Fairfield, USA; 6 Internal Medicine, Piedmont Athens Regional Medical, Athens, USA; 7 General Practice, El Demerdash Hospital, Cairo, EGY; 8 Research, California Institute of Behavioral Neurosciences & Psychology, Fairfield, USA

**Keywords:** propranolol, carvedilol, hepatic venous pressure, variceal bleeding, portal hypertension, liver cirrhosis

## Abstract

The most common cause of portal hypertension is liver cirrhosis. Portal hypertension causes many complications in cirrhotic patients; a significant complication is the formation of varices and the subsequent life-threatening variceal bleeding due to elevated portal venous pressures. Hepatic venous pressure gradient (HVPG) is the gold standard for measuring portal hypertension and guides management. Pharmacological treatments lower the HVPG, preventing the progression of varices and subsequent variceal bleeding. The pharmacological treatments frequently used in primary and secondary prophylaxis of a variceal bleed are nonselective beta (β)-adrenergic blockers. Propranolol was the first nonselective β-adrenergic blocker used for lowering HVPG and has been well studied. However, in the past decade, clinical trials have shown that carvedilol has been more effective. This study aims to establish whether carvedilol is more effective than propranolol in reducing the hepatic venous pressure gradient and decreasing the risk of variceal bleeding in adult cirrhotic patients. A systematic review has been conducted to gather relevant clinical trials comparing drugs and their effects on HVPG. Four databases: PubMed (Medical Literature Analysis and Retrieval System Online (MEDLINE)), Google Scholar, the Cochrane Library, and ScienceDirect, were analyzed, and records from January 1, 1999, to January 1, 2023, were chosen. There were a total of 1,235 potentially eligible records across the four databases. Using the eligibility criteria for this systematic review, seven studies of 533 patients were included. Across all seven clinical trials, it was found that carvedilol reduced HVPG more than propranolol and decreased the risk of variceal bleeding in adult cirrhotic patients.

## Introduction and background

Liver cirrhosis and its complications

Liver cirrhosis is a severe and potentially life-threatening condition, with an estimated global prevalence of 0.27% to 0.45% and a mortality rate of 14.6% to 44.2% over five years, depending on the severity of the disease and the underlying cause [1]. Liver cirrhosis is a chronic liver disease characterized by scar tissue formation, known as fibrosis, that replaces healthy liver tissue and leads to progressive liver dysfunction. Liver cirrhosis has many etiologies, including chronic alcohol use, non-alcoholic fatty liver disease, viral hepatitis B and C, autoimmune hepatitis, and genetic disorders such as hemochromatosis and Wilson's disease [2]. Liver cirrhosis can be classified as either compensated or decompensated based on the severity of the liver dysfunction and complications. Compensated cirrhosis refers to a stage of liver disease with liver scarring. However, the liver can still perform its essential functions, and no significant complications are present. Compensated cirrhosis patients may have no symptoms or mild symptoms such as fatigue, weakness, or mild jaundice. Patients with compensated cirrhosis need management as it can progress to decompensated cirrhosis. Decompensated liver cirrhosis is a more advanced stage of liver disease where the liver can no longer perform its essential functions, causing significant complications such as variceal bleeding, ascites, jaundice, and hepatic encephalopathy. Without treatment, the prognosis of decompensated cirrhosis is generally poor, with a high risk of liver failure and death. The diagnosis of liver cirrhosis involves a combination of clinical evaluation, laboratory tests, imaging studies, and, in some cases, a liver biopsy. The treatment approach for compensated and decompensated cirrhosis varies depending on the underlying cause, severity of liver dysfunction, and complications [2-5].

Portal hypertension and variceal bleeding 

Portal hypertension is an increase in hepatic vascular resistance to portal blood flow. The most common cause of portal hypertension is liver cirrhosis. It is defined by an increased portal venous pressure gradient above 5 millimeters of mercury (mmHg) [6]. The pathophysiology of portal hypertension in cirrhotic patients includes the gross structural changes that occur following the diffuse fibrosis and the formation of nodules of regenerating hepatocytes, remodeling and capillarization of the hepatic sinusoids, endothelial dysfunction, and the disorder of paracrine interactions between damaged hepatocytes, sinusoidal endothelial cells (SEC), Kupffer cells, and activated hepatic stellate cells (HSC) of the liver [7-8]. Other factors, such as splanchnic hyperemia, the formation of collateral circulation and established hyperdynamic circulation, vascular remodeling, and endothelial dysfunction, all contribute to the progression of portal hypertension [9]. Portal hypertension causes significant complications, including variceal bleeding, ascites, hepatic encephalopathy, hepatorenal renal syndrome, portal hypertensive gastropathy, and spontaneous bacterial peritonitis. This systematic review focuses on the specific complications of varices and variceal bleeding. Varices are enlarged and tortuous blood vessels caused by obstructed blood flow through the liver, causing the blood to be backed up and rerouted to smaller veins in the esophagus, stomach, and rectum, which are known as esophageal, gastric, and rectal varices, respectively. Varices usually cannot handle these increased pressures, leading to their dilation and eventual rupture. The most common type of varices are esophageal varices, and they must be managed as an esophageal variceal bleed can be life-threatening [6-9]. The most common cause of mortality in cirrhotic patients is variceal bleeding, and the mortality rate is 20% [10]. Hence, it is of utmost importance to lower portal hypertension to prevent varices from progressing and leading to a life-threatening bleed [11].

Hepatic venous pressure

The gold standard method of measuring portal hypertension is the hepatic venous pressure gradient (HVPG), which is used to assess the severity of portal hypertension and guide its management. The HVPG is calculated by measuring the difference between the wedged hepatic venous pressure (WHVP) and the free hepatic venous pressure (FHVP). To measure the WHVP, a catheter is inserted into the hepatic vein and advanced to a hepatic venous branch, which is wedged to occlude the venous flow temporarily. The pressure measured at this point represents the WHVP. To measure the FHVP, the catheter is withdrawn close to the inferior vena cava (IVC), around 1 cm to 2 cm inside the hepatic vein. The pressure measured at this point represents the FHVP. The difference between the WHVP and the FHVP is the HVPG, which reflects the pressure gradient between the portal vein and the inferior vena cava. An HVPG of less than or equal to 5 mmHg is normal; 6-9 mmHg is mild portal hypertension; 10-19 mmHg is moderate portal hypertension; and ≥20 mmHg indicates severe portal hypertension [12-15]. In patients with liver cirrhosis and portal hypertension, the target HVPG is less than 12 mmHg, or 20% of the baseline, as it has been shown to reduce the risk of variceal bleeding and mortality [7, 16].

Pharmacological management to decrease portal hypertension in cirrhotic patients

There are pharmacological and interventional approaches to reducing portal hypertension, and the focus of this systematic review will be based on the pharmacological approach. Pharmacological therapy aims at decreasing hepatic vascular resistance by preserving or increasing portal blood flow and correcting hyperdynamic circulation and pathological angiogenesis while striving to reduce the hepatic venous pressure gradient to less than 12 mmHg, or 20% of the baseline [7]. Patients responding to pharmacological treatment are called "good responders," in which a patient achieves a reduction in hepatic venous pressure gradient (HVPG) of at least 20% or to an absolute value below 12 mmHg [17]. Most drugs currently used to lower portal hypertension are splanchnic vasoconstrictors, whose effect is based on reducing splanchnic blood flow and hyperdynamic circulation. Drugs of use include vasopressin derivatives, somatostatin, long-acting somatostatin analogs, nonselective beta (β)-adrenergic blockers alone and combined with vasodilators, and other drugs currently under investigation [7]. This systematic review focuses on nonselective β-adrenergic blockers, especially propranolol and carvedilol. Nonselective β-adrenergic blockers are used for primary and secondary variceal bleeding prophylaxis.

Primary prophylaxis prevents a variceal bleed in patients with a high risk of a variceal bleed [17]. The recommendations for primary prophylaxis of esophageal varices depend on multiple risk factors. The most critical risk factor is variceal size. Varices can be classified as small, medium, or large. The latter classification method has often been recommended, with a suggested cutoff diameter of 5 millimeters (mm). The risk of bleeding from small varices has been reported to be 7%, whereas the risk from large varices increases to 30% [18]. Secondary prophylaxis prevents the patient from rebleeding after experiencing a first episode of variceal bleeding [17]. Patients who survive and recover from an episode of acute variceal bleeding are at high risk of rebleeding and death, which is 60% and 33% in the first year, respectively [19- 20].

Nonselective β-adrenergic blockers are the drugs of choice for preventing bleeding from esophageal varices [13]. This is due to their effects targeting the pathogenesis of portal hypertension in cirrhotic patients, including blocking β2-adrenergic vascular receptors, which causes unopposed alpha 1 (α1)-adrenergic activity and results in splanchnic vasoconstriction and the reduction of portal inflow. They also work to block β1-adrenergic cardiac receptors, reducing cardiac output and thus improving hyperdynamic circulation. Additionally, they reduce azygos blood flow and variceal pressure. An additional benefit found was that they reduce the risk of bacterial translocation by decreasing intestinal transit time, which has been related to decreased bacterial overgrowth [7]. The first nonselective β-adrenergic blocker introduced into clinical practice for treating portal hypertension in cirrhotic patients was propranolol. Propranolol’s impact on the portal and systemic hemodynamics is well studied. It is established that propranolol can decrease HVPG by 10%-31%, azygal blood flow by 29%-47%, cardiac output by 10%-31%, mean arterial pressure by 0%-14%, and hepatic blood flow by 0%-39% [21]. In the past decade, several studies have used carvedilol, another nonselective β-adrenergic blocker agent, instead of propranolol to treat cirrhotic patients with portal hypertension. This drug has weak anti-α1-adrenergic activity, which is thought to contribute to reducing portal hypertension in cirrhotic patients. The α1-adrenergic receptors are responsible for mediating a vasoconstrictive response. There is increased activity of the sympathetic nervous system in portal hypertension, which results in vasoconstriction of the splanchnic vasculature, elevating portal pressure. By blocking the alpha (α)1-adrenergic receptors, carvedilol can cause vasodilation of the splanchnic vasculature and reduce portal pressure [22-23]. This systematic review investigates whether carvedilol is more effective than propranolol in reducing the hepatic venous pressure gradient and preventing variceal bleeding in adult cirrhotic patients. This is an important study area since approximately 60%-80% of cirrhotic patients develop varices, and the risk of variceal bleeding is up to 25%-35% [24]. Due to the high prevalence of varices in cirrhotic patients and their devastating consequences, it is important to establish which pharmacological approach is more effective in reducing mortality and improving the quality of life in cirrhotic patients.

## Review

Methods

The findings of this systematic review implement the Preferred Reporting Items for Systematic Reviews and Meta-Analyses (PRISMA) 2020 guidelines [25].

Eligibility Criteria

The Population, Intervention, Control, and Outcomes (PICO) criteria were used as the primary framework for the eligibility criteria. The literature selected for this systematic review is based on all the components of the PICO criteria, as follows:

Population: Selection of studies with adult cirrhotic patients ≥19 years old from all ethnicities and genders; Intervention: Carvedilol for reducing HVPG and lowering the risk of a variceal bleed; Comparison: Propranolol for reducing HVPG and lowering the risk of a variceal bleed; and Outcomes: Studies displaying evidence of which drug, carvedilol or propranolol, lowered HVPG and reduced the risk of a variceal bleed

Inclusion and Exclusion Criteria

The inclusion criteria in this systematic review included studies investigating whether carvedilol is more effective than propranolol in reducing the hepatic venous pressure gradient and decreasing the risk of variceal bleeding in adult cirrhotic patients. There were no gender or ethnic restrictions on the population of the studies. Papers of all languages with English translations, free full-text articles published between 1999 and 2023, randomized controlled trials, non-randomized control trials, meta-analyses, literature, and systematic reviews were included in the study. Case studies, case reports, gray literature, animal studies, and editorials were excluded from this systematic review.

Search Strategy

The online search used PubMed, Google Scholar, the Cochrane Library, and ScienceDirect. Keywords that have been utilized for the websites mentioned above include "carvedilol," "propranolol," "non-selective adrenergic beta-agonists," "portal hypertension," "hepatic venous pressure gradient," "esophageal and gastric varices", "gastrointestinal hemorrhage", and "liver cirrhosis." The Medical Subject Headings Search (MeSH) targeted the keywords presented in this systematic review. The Boolean method combined the keywords to search the databases mentioned above uniformly; MeSH concepts, key terms, and search results from each database are shown in Tables 1-2.

**Table 1:** Databases and search strategies MeSH: Medical Subject Headings

MeSH concepts	Database	Number of studies (with filters)	Date and time (time zone: Eastern Standard Time)
Carvedilol OR Non-selective Adrenergic beta-Antagonists OR ("Carvedilol/administration and dosage"[Mesh] OR "Carvedilol/adverse effects"[Mesh] OR "Carvedilol/analysis"[Mesh] OR "Carvedilol/blood"[Mesh] OR "Carvedilol/chemistry"[Mesh] OR "Carvedilol/classification"[Mesh] OR "Carvedilol/metabolism"[Mesh] OR "Carvedilol/pharmacokinetics"[Mesh] OR "Carvedilol/pharmacology"[Mesh] OR "Carvedilol/therapeutic use"[Mesh] OR "Carvedilol/toxicity"[Mesh] ) Filters: Free full text, 1999-2013, Humans, Adult: 19+ years	PubMed	169	03/06/2023 12:02 pm
Propranolol OR Non-selective Adrenergic beta-Antagonists OR ("Propranolol/administration and dosage"[Majr] OR "Propranolol/adverse effects"[Majr] OR "Propranolol/analysis"[Majr] OR "Propranolol/blood"[Majr] OR "Propranolol/chemistry"[Majr] OR "Propranolol/metabolism"[Majr] OR "Propranolol/pharmacokinetics"[Majr] OR "Propranolol/pharmacology"[Majr] OR "Propranolol/therapeutic use"[Majr] OR "Propranolol/toxicity"[Majr] ) Filters: Free full text, 1999-2013, Humans, Adult: 19+ years	PubMed	182	03/06/2023 12:11 pm
Hepatic venous pressure gradient OR Portal hypertension OR ( "Hypertension, Portal/blood"[Majr] OR "Hypertension, Portal/classification"[Majr] OR "Hypertension, Portal/complications"[Majr] OR "Hypertension, Portal/diagnosis"[Majr] OR "Hypertension, Portal/diagnostic imaging"[Majr] OR "Hypertension, Portal/drug therapy"[Majr] OR "Hypertension, Portal/etiology"[Majr] OR "Hypertension, Portal/mortality"[Majr] OR "Hypertension, Portal/physiopathology"[Majr] OR "Hypertension, Portal/prevention and control"[Majr] OR "Hypertension, Portal/therapy"[Majr] ) Filters: Free full text, 1999-2013, Humans, Adult: 19+ years	PubMed	158	03/06/2023 12:12 pm
Varices OR Esophageal and Gastric Varices OR ("Esophageal and Gastric Varices/blood"[Majr] OR "Esophageal and Gastric Varices/complications"[Majr] OR "Esophageal and Gastric Varices/drug therapy"[Majr] OR "Esophageal and Gastric Varices/physiopathology"[Majr] OR "Esophageal and Gastric Varices/prevention and control"[Majr] OR "Esophageal and Gastric Varices/therapy"[Majr] ) Filters: Free full text, 1999-2013, Humans, Adult: 19+ years	PubMed	424	03/06/2023 12:13 pm
Variceal Bleeding OR Gastrointestinal Hemorrhage OR ("Gastrointestinal Hemorrhage/blood"[Majr] OR "Gastrointestinal Hemorrhage/classification"[Majr] OR "Gastrointestinal Hemorrhage/complications"[Majr] OR "Gastrointestinal Hemorrhage/drug therapy"[Majr] OR "Gastrointestinal Hemorrhage/etiology"[Majr] OR "Gastrointestinal Hemorrhage/mortality"[Majr] OR "Gastrointestinal Hemorrhage/physiopathology"[Majr] OR "Gastrointestinal Hemorrhage/prevention and control"[Majr] OR "Gastrointestinal Hemorrhage/therapy"[Majr] ) Filters: Filters: Free full text, 1999-2013, Humans, Adult: 19+ years	PubMed	372	03/06/2023 12:15 pm
Cirrhotic patients OR Liver Cirrhosis OR ("Liver Cirrhosis/blood"[Mesh] OR "Liver Cirrhosis/classification"[Mesh] OR "Liver Cirrhosis/complications"[Mesh] OR "Liver Cirrhosis/drug therapy"[Mesh] OR "Liver Cirrhosis/mortality"[Mesh] OR "Liver Cirrhosis/physiopathology"[Mesh] OR "Liver Cirrhosis/prevention and control"[Mesh] OR "Liver Cirrhosis/therapy"[Mesh] ) Filters: Free full text, 1999-2013, Humans, Adult: 19+ years	PubMed	754	03/06/2023 12:17 pm
Carvedilol OR Non- Selective Adrenergic beta-Antagonists OR ("Carvedilol/administration and dosage"[Mesh] OR "Carvedilol/adverse effects"[Mesh] OR "Carvedilol/analysis"[Mesh] OR "Carvedilol/blood"[Mesh] OR "Carvedilol/chemistry"[Mesh] OR "Carvedilol/classification"[Mesh] OR "Carvedilol/metabolism"[Mesh] OR "Carvedilol/pharmacokinetics"[Mesh] OR "Carvedilol/pharmacology"[Mesh] OR "Carvedilol/therapeutic use"[Mesh] OR "Carvedilol/toxicity"[Mesh] ) AND Propranolol OR Non- selective Adrenergic beta-Antagonists OR ("Propranolol/administration and dosage"[Majr] OR "Propranolol/adverse effects"[Majr] OR "Propranolol/analysis"[Majr] OR "Propranolol/blood"[Majr] OR "Propranolol/chemistry"[Majr] OR "Propranolol/metabolism"[Majr] OR "Propranolol/pharmacokinetics"[Majr] OR "Propranolol/pharmacology"[Majr] OR "Propranolol/therapeutic use"[Majr] OR "Propranolol/toxicity"[Majr] ) AND Varices OR Esophageal and Gastric Varices OR ("Esophageal and Gastric Varices/blood"[Majr] OR "Esophageal and Gastric Varices/complications"[Majr] OR "Esophageal and Gastric Varices/drug therapy"[Majr] OR "Esophageal and Gastric Varices/physiopathology"[Majr] OR "Esophageal and Gastric Varices/prevention and control"[Majr] OR "Esophageal and Gastric Varices/therapy"[Majr] ) AND Variceal Bleeding OR Gastrointestinal Hemorrhage OR ("Gastrointestinal Hemorrhage/blood"[Majr] OR "Gastrointestinal Hemorrhage/classification"[Majr] OR "Gastrointestinal Hemorrhage/complications"[Majr] OR "Gastrointestinal Hemorrhage/drug therapy"[Majr] OR "Gastrointestinal Hemorrhage/etiology"[Majr] OR "Gastrointestinal Hemorrhage/mortality"[Majr] OR "Gastrointestinal Hemorrhage/physiopathology"[Majr] OR "Gastrointestinal Hemorrhage/prevention and control"[Majr] OR "Gastrointestinal Hemorrhage/therapy"[Majr] ) AND cirrhotic patients OR Liver Cirrhosis OR ("Liver Cirrhosis/blood"[Mesh] OR "Liver Cirrhosis/classification"[Mesh] OR "Liver Cirrhosis/complications"[Mesh] OR "Liver Cirrhosis/drug therapy"[Mesh] OR "Liver Cirrhosis/mortality"[Mesh] OR "Liver Cirrhosis/physiopathology"[Mesh] OR "Liver Cirrhosis/prevention and control"[Mesh] OR "Liver Cirrhosis/therapy"[Mesh] ) Filters: Free full text, 1999-2013, Humans, Adult: 19+ years	PubMed	722	03/06/2023 12:28 pm

**Table 2:** Databases and search strategies

Key terms	Database	Number of studies (with filters)
Carvedilol AND Propranolol AND Esophageal and Gastric Varices	Google Scholar	1,320
	Cochrane Library	19
	ScienceDirect	115
Carvedilol AND Esophageal and Gastric Varices	Google Scholar	1,710
	Cochrane Library	46
	ScienceDirect	135
Propranolol AND Esophageal and Gastric Varices	Google Scholar	6,420
	Cochrane Library	185
	ScienceDirect	426
Non-selective Adrenergic Beta-Antagonists AND Esophageal and Gastric Varices	Google Scholar	41
	Cochrane Library	20
	ScienceDirect	93
Non-selective Adrenergic Beta-Antagonists AND Gastrointestinal Hemorrhage	Google Scholar	240
	Cochrane Library	19
	ScienceDirect	812
Carvedilol AND Portal Hypertension	Google Scholar	5,240
	Cochrane Library	78
	ScienceDirect	415
Propranolol AND Portal Hypertension	Google Scholar	14,300
	Cochrane Library	251
	ScienceDirect	1,090
Carvedilol AND Hepatic Venous Pressure Gradient	Google Scholar	10,100
	Cochrane Library	34
	ScienceDirect	286
Propranolol AND Hepatic Venous Pressure Gradient	Google Scholar	16,600
	Cochrane Library	92
	ScienceDirect	587
Carvedilol AND Propranolol AND Hepatic Venous Pressure Gradient	Google Scholar	3,850
	Cochrane Library	20
	ScienceDirect	178
Carvedilol AND Propranolol AND Hepatic Venous Pressure Gradient AND Esophageal and Gastric Varices AND Gastrointestinal Hemorrhage	Google Scholar	863
	Cochrane Library	3
	ScienceDirect	59
Carvedilol AND Gastrointestinal Hemorrhage	Google Scholar	4,100
	Cochrane Library	27
	ScienceDirect	507
Propranolol AND Gastrointestinal Hemorrhage	Google Scholar	12,900
	Cochrane Library	168
	ScienceDirect	1,397
Liver Cirrhosis AND Carvedilol	Google Scholar	5,900
	Cochrane Library	106
	ScienceDirect	502
Liver Cirrhosis AND Propranolol	Google Scholar	14,900
	Cochrane Library	311
	ScienceDirect	1,273

Data Selection and Extraction

Two researchers (LD and MT) independently selected and extracted relevant studies. Disagreements over eligibility between the two researchers were resolved by discussing the study design, intervention implemented, outcomes measured, and relevance to inclusion and exclusion criteria. A third researcher (TS) was solicited when a uniform consensus could not be reached. Across all databases, there were a total of 1,235 potential eligible records. Based on the eligibility criteria, a total of seven studies consisting of 533 patients formed the basis of this review. The studies included seven randomized controlled trials. From the studies included, the following data were retrieved: (i) the surname of the principal author and the publication date; (ii) a study overview (i.e., study design, sample size, and duration of follow-up); and (iii) general features of the study population (intervention, dose and frequency, HVPG measurement, and the respective p-values and outcomes).

Study Quality Appraisal

Each study used in this systematic review was assessed for the potential risk of bias. Clinical trials were evaluated using the revised Cochrane risk of bias 2 (RoB 2) tool. With the latest Cochrane RoB 2 tool, each randomized controlled trial was assessed for potential biases based on five risks of bias. Each risk of bias was scored as either low, high, or moderate. Subsequently, the overall risk of bias was also reported as low, moderate, or high [26]. Table 3 shows the results of the revised Cochrane RoB 2 tool.

**Table 3:** Assessment of clinical trials using the revised Cochrane Risk of Bias 2 tool RoB: risk of bias; LR: low risk; MR: moderate risk; HR: high risk

First author (Year)	Random allocation	Intervention non-adherence	Incomplete results	Inadequate assessment of the outcomes	Selective reporting	Final RoB judgment
Agarwala et al., 2011 [27]	MR	HR	MR	LR	HR	HR
Bañares et al., 1999 [28]	LR	LR	HR	LR	MR	HR
Bañares et al., 2002 [29]	LR	MR	HR	LR	HR	HR
De et al., 2002 [30]	LR	LR	HR	LR	LR	HR
Gupta et al., 2016 [31]	LR	HR	HR	LR	LR	HR
Sharma et al., 2020 [32]	LR	LR	LR	LR	LR	LR
Hobloth et al., 2012 [33]	LR	LR	HR	LR	LR	HR

Results

The various search strategies across the databases identified 1,235 records. Out of 1,235 records, 726 originated from PubMed (Medical Literature Analysis and Retrieval System Online (MEDLINE)), 492 from Google Scholar, two from the Cochrane Library, and 15 from ScienceDirect. No other resource was used. A total of three duplicates were removed online using EndNote before screening the articles. The remaining 1,232 records were thoroughly screened for relevance based on titles and abstracts, after which 1,000 records were excluded due to their irrelevance to the topic, research objectives, inclusion, and exclusion criteria. Hence, 232 articles were sought for retrieval, and after thoroughly checking again for relevance to the research topic, inclusion and exclusion criteria, and free full texts, a further 213 were removed.

**Figure 1:**
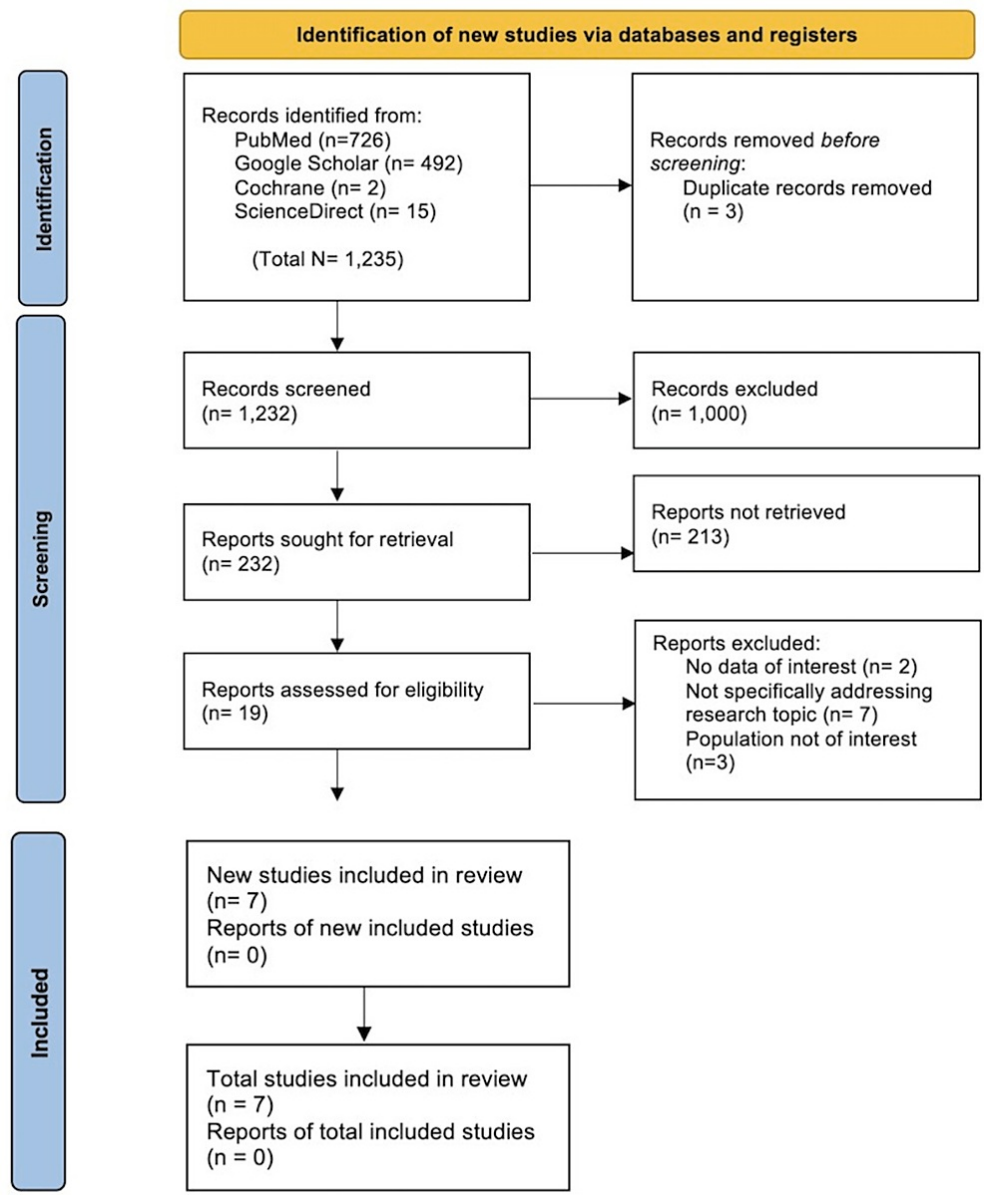
A Preferred Reporting Items for Systematic Reviews and Meta-Analyses (PRISMA) flow diagram to depict the selection of studies for the systematic review N: number

A total of 19 reports were assessed for eligibility, and 12 were removed. Two reports were removed as they contained no data of interest; seven were removed as they did not address the research topic; and three were removed because they needed more population of interest. Therefore, seven reports were included in this systematic review and all seven were randomized control trials. The complete PRISMA flow diagram is shown in Figure 1.

Table 4 demonstrates the baseline characteristics and outcomes of the randomized control trials chosen.

**Table 4:** Baseline characteristics and outcomes of the studies RCT: randomized control trial; C: carvedilol; P: propranolol; HVPG: hepatic venous pressure gradient; P.O: per oral; NA: not available *** It reported different numbers of patients according to measurements.

Author, Year	Study design	Sample size	Intervention	Dose and frequency	Follow-up	HVPG measurement	Outcomes
Agarwala et al., 2011 [27]	RCT	Total 102; C: 54; P: 48	C vs. P	NA	At or after 6 months	NA	Carvedilol was more effective in preventing variceal bleeding (8.33% vs 31.25; P = 0.0284 ).
Bañares et al., 1999 [28]	RCT	Total 166; C: 14; P: 145; Placebo: 7	C vs. P vs. placebo	C: 25 mg P.O., P: 0.15 mg/kg I.V. followed by a continuous infusion of 0.2 mg/kg/h	60 min	Carvedilol markedly reduced HVPG, from 19.5 ± 1.3 to 15.4 ± 1 mm Hg (P < .0001). This HVPG reduction was greater than after propranolol (−20.4 ± 2 vs. −12.7 ± 2%, P < .05).	Carvedilol decreased HVPG to greater than 20% of baseline values or to ≤12 mm Hg in a greater proportion of patients (64% vs. 14%, P < .05).
Bañares et al., 2002 [29]	RCT	Total 51; C: 26; P: 25	C vs. P	C: 31 ± 4 mg/day P.O. (range, 12.5–50), P: 73 ± 10 mg/day P.O. (range, 10–160)	11 ± 4.1 weeks	Carvedilol caused a greater decrease in HVPG than propranolol (-19 +/- 2% vs. -12 +/- 2%; P < .001)	The proportion of patients achieving an HVPG reduction >/=20% or =12 mm Hg was greater after carvedilol (54% vs. 23% P < .05)
De et al., 2002 [30]	RCT	Total 69; C Acute: 18; P Acute: 18; C Chronic: 15-17 ***; P Chronic: 14-16***	C vs. P	Acute: C: 25 mg P.O., P: 80 mg P.O.; Maintenance: C: 6.25 mg P.O. twice daily, P: 40 mg P.O. twice daily	Acute: 90 min Chronic: 1 week	HVPG reduction (percent) by carvedilol was not superior to that by propranolol either acutely (27.67 +/- 31.49 compared to 22.98 +/- 27.40, P = 0.6) or after 7 days (28.2 +/- 29.05 compared to 23.25 +/- 20.15, P = 0.6).	Carvedilol slightly reduced HVPG more than propranolol.
Gupta et al., 2016 [31]	RCT	Total 59; C: 30; P: 29	C vs. P	C: 3.125 mg two times per day, P: 40 mg per day	1 month	Higher incidence of decreased HVPG > 20 % / <12 mmHg was documented in the carvedilol group compared to the propranolol group (75% vs 50%; p = 0.04).	Carvedilol is more effective than propranolol in reducing portal pressure in cirrhotic patients with esophageal bleed.
Sharma et al., 2020 [32]	RCT	Total 48; C: 25; P: 23	C vs. P	NA	6 years	More number of patients on carvedilol had HVPG response when compared with those taking propranolol (72% carvedilol versus 47.8% propranolol, p = 0.047)	Carvedilol had a significant HVPG response when compared to propranolol.
Hobloth et al., 2012 [33]	RCT	Total 38; C: Acute and Chronic: 21; P: Acute and Chronic: 17	C vs. P	C acute: 6.25 mg; P Acute: 80 mg; C Chronic: 14 mg; P Chronic: 122 mg	Acute: 90min; Chronic: 92.7±13.6 days	HVPG decreased by 19.3 ± 16.1% (p < 0.01) and by 12.5 ± 16.7% (p < 0.01) in the carvedilol and propranolol groups, respectively,	Carvedilol reduced HVPG more than propranolol however no significant difference between treatment regimens (p = 0.21) was noted in this study.

Discussion

Clinical Trial Results

In the seven clinical trials that were chosen for this systematic review, all have displayed that carvedilol reduced HVPG more than propranolol, hence reducing the risk of variceal bleeding. Agarwala et al., in a randomized controlled trial, found that of the 36 patients that received carvedilol, only three had a first variceal bleed during the six-month follow-up. Of 32 patients receiving propranolol, 10 had first variceal bleeding during the six-month follow-up (8.33% vs. 31.25%; p-0.0284). Regarding rebleeding after the first variceal bleed, out of 21 patients taking carvedilol, three had rebleeding, compared to 10 out of 22 in the propranolol group (14.28% vs. 45.45%; p-0.0452). Furthermore, this study highlights that carvedilol is more effective in lowering the risk of variceal bleeding than propranolol and can be used as both primary and secondary prophylaxis for variceal bleeding [27]. In Bañares et al.'s study, 35 cirrhotic patients were randomly allocated to receive carvedilol, propranolol, or placebo. They had hemodynamic measurements before and after administering the drugs, and the follow-up was an acute period of 60 minutes. It was found that carvedilol markedly reduced HVPG from 19.5 +/- 1.3 mm Hg to 15.4 +/- 1 mm Hg (p <.0001) compared with propranolol (-20.4 +/- 2 vs. -12.7 +/- 2%, p <.05). Moreover, carvedilol patients had a good response to the medication (decreased HVPG greater than 20% of baseline values or to </=12 mm Hg) in a greater proportion of patients (64% vs. 14%, p <.05) compared to propranolol. Regarding hepatic and azygous blood flows, both drugs caused similar reductions. This suggests that the greater HVPG decrease caused by carvedilol was because of its ability to reduce hepatic and portal collateral resistance [28]. In Bañares et al.'s study, 51 patients were randomly assigned to receive either carvedilol or propranolol. The hemodynamic and renal functions were assessed at baseline and after 11.1 +/- 4.1 weeks. Carvedilol caused a greater decrease in HVPG than propranolol (-19 +/- 2% vs. -12 +/- 2%; p <.001). The proportion of patients achieving an HVPG reduction >/=20% or </=12 mm Hg was greater after carvedilol (54% vs. 23%; p <.05) [29]. In a study by De et al., 36 cirrhotic patients were randomized into two groups, one receiving carvedilol and one receiving propranolol. The study measured whether patients achieved a good response after pharmacological treatment. The HVPG was measured before and after 90 minutes of oral administration of either carvedilol 25 mg or propranolol 80 mg and again seven days after carvedilol 12.5 mg daily or propranolol 80 mg daily, respectively. With carvedilol, 11/18 (61.1%) and 11/17 (64.7%) patients responded acutely and after seven days, respectively, while 9/18 (50%) and 10/16 (62.5%) did so with propranolol. However, it was found that HVPG reduction by carvedilol was not superior to that by propranolol either acutely (27.67 +/- 31.49 compared to 22.98 +/- 27.40, p = 0.6) or after seven days (28.2 +/- 29.05 compared to 23.25 +/- 20.15, p = 0.6). However, the study stated that the population was consistent with only Indian patients and that more research with a broader population might display more significance [30].

Gupta et al. studied the effect of carvedilol versus propranolol on HVPG in 59 cirrhotic patients after having their first variceal bleeding (the variceal bleed was treated by somatostatin followed by endoscopic variceal ligation in all patients in the study). Patients were followed up after one month, and it was found that HVPG was reduced significantly in both groups (p = 0.001); the number of HVPG responders was significantly higher in the carvedilol group (22/29) as compared to the propranolol group (14/28), p = 0.04. Hence, carvedilol effectively reduces portal pressure in cirrhotic patients with esophageal bleeding [31]. In Sharma et al.'s study, 48 patients were split into two groups taking either carvedilol or propranolol and were followed up for six years. Patients taking carvedilol had a greater HVPG response when compared with those taking propranolol (72% carvedilol vs. 47.8% propranolol, p = 0.047). It was found that the rate of variceal rebleeding at the one-year and three-year follow-ups was lower in carvedilol (16.0% and 24.0%) versus 8.9% and 36.7% for propranolol (p = 0.457) [32]. In Hobloth et al.'s study, 38 cirrhotic patients were put into either carvedilol or a propranolol group and had both acute (90 minutes) and chronic (90 days) follow-ups. It was found that carvedilol decreased HVPG more than propranolol; however, it was of little significance. Hepatic venous pressure gradient decreased by 19.3 ± 16.1% (p < 0.01) and by 12.5 ± 16.7% (p < 0.01) in the carvedilol and propranolol groups, respectively. This randomized study demonstrated that carvedilol is as effective as propranolol on HVPG after long-term administration [33]. Therefore, the clinical trials demonstrate that carvedilol does reduce HVPG more than propranolol and prevents the risk of variceal bleeding in adult cirrhotic patients. The anti-α1-adrenergic activity of carvedilol contributes to its beneficial effects in reducing portal hypertension. As mentioned earlier, blocking the α1-adrenergic receptors causes vasodilation of the splanchnic vasculature, which reduces portal pressure [10].

Carvedilol’s Effects on Hemodynamics Other than HVPG

Mean arterial pressure (MAP) is the average blood pressure during a single cardiac cycle. The hemodynamic target in cirrhotic patients is a systolic blood pressure above 90 mmHg with a pulse above 55 beats per minute. In patients with decompensated cirrhosis, carvedilol can be used at low doses if the systolic blood pressure is above 90 mmHg [34]. Nonselective β-adrenergic blockers, such as propranolol and carvedilol, lower MAP by blocking beta-1 adrenergic receptors in the heart and peripheral vasculature. This causes a reduction in heart rate, cardiac output, and peripheral vascular resistance, which collectively decrease blood pressure. Blocking beta-1 receptors in the heart decreases heart rate and contractility, reducing cardiac output. Combining the effects of reduced cardiac output and peripheral vascular resistance leads to decreased MAP. Nonselective β-adrenergic blockers also affect the renin-angiotensin-aldosterone system (RAAS), lowering MAP. This is done by blocking beta-1 receptors in the juxtaglomerular cells of the kidneys, causing a reduction in the secretion of renin, which decreases angiotensin II production and aldosterone secretion, both of which contribute to vasoconstriction and sodium and water retention, respectively. Overall, nonselective β-adrenergic blockers lower MAP through a combination of decreased cardiac output, reduced peripheral vascular resistance, and modulation of the RAAS [35-36].

Studies have shown that reducing MAP can lead to a reduction in HVPG, reducing the risk of variceal bleeding and improving overall outcomes in cirrhotic patients. Tripathi et al.'s randomized controlled trial of 213 cirrhotic patients with portal hypertension found that using nonselective β-adrenergic blockers lowered MAP and was associated with a significant reduction in HVPG and a lower risk of variceal bleeding compared to placebo [37]. Lee et al. performed a study of 138 cirrhotic patients and found that reducing MAP can decrease portal vein diameter, which indicates a reduction in portal hypertension [38]. Several studies have shown that carvedilol decreases MAP more than propranolol in cirrhotic patients. In a study by Bañares et al., carvedilol caused a more significant decrease in mean arterial pressure when compared to propranolol (-23.1 vs. -11%, p <.05). However, it was found that propranolol caused more significant reductions in heart rate and cardiac output than carvedilol. Hence, this study highlights that carvedilol has a greater portal hypotensive effect than propranolol in patients with cirrhosis, suggesting a more significant therapeutic potential [28]. In the study by Bañares et al., carvedilol caused a significant decrease in mean arterial pressure (MAP) compared to propranolol (-11 +/- 1% vs. -5 +/- 3%; p =.05), further highlighting carvedilol’s greater portal hypotensive effect [30]. Another study by Gupta et al. demonstrated that carvedilol decreased heart rate and MAP more than propranolol. The percentage decrease in MAP was significantly higher in the carvedilol group than in the propranolol group (p = 0.04) [31]. Lowering the MAP in cirrhotic patients needs to be carefully monitored, as it can have adverse effects. One of the main concerns about lowering the MAP in cirrhotic patients is arterial hypotension, which causes decreased renal perfusion and hepatorenal syndrome [39]. Hepatorenal syndrome (HRS) is a rapidly progressive form of acute renal failure that occurs in cirrhotic patients and is characterized by a reduction in glomerular filtration rate (GFR) and renal plasma flow [40]. Studies have shown that excessive reductions in MAP in cirrhotic patients can lead to acute kidney injury and increased mortality [41]. However, it is worth noting that Tripathi et al. found that carvedilol reduced both HVPG and systemic vascular resistance, which decreased cardiac output and MAP. However, this decrease in cardiac output was not associated with an increased incidence of hepatorenal syndrome or hepatic encephalopathy [42]. Another concern about lowering MAP in cirrhotic patients is the exacerbation of the underlying cardiovascular disease, as cirrhotic patients are at an increased risk of cardiovascular events [15].

Carvedilol's Side Effect Profile Compared with Propranolol 

While carvedilol seems like an effective alternative to propranolol, it is important to discuss its side effect profile. The two main concerns about the clinical use of carvedilol in cirrhotic patients include hypotension and new-onset or worsening ascites. Hypotension in cirrhotic patients using carvedilol would manifest as fatigue, weakness, dizziness, and orthostatic hypotension. Orthostatic hypotension was reported by Bañares et al., where 14/65 patients taking carvedilol experienced orthostatic hypotension compared with the 9/60 propranolol-treated patients. This calls for caution when used in clinical practice [28]. In the study by Agarwala et al., the incidence of adverse events during the study was similar in the two groups except for a higher but statistically insignificant (p > 0.05) incidence of hypotension in the carvedilol group [27]. De et al. found that one carvedilol patient with ascites had symptomatic orthostatic hypotension, followed by oliguria requiring treatment withdrawal and management [30]. However, it is essential to note that the hypotensive effect of carvedilol was markedly reduced by starting with a very low dose (e.g., 6.25 mg/day), careful titration, and stepwise increases every four to seven days [43, 44].

Regarding worsening ascites, Bañares et al. noted a significant increase in plasma volume and body weight in cirrhotic patients receiving carvedilol and not propranolol. Additionally, an increase in diuretic dosage was needed in seven carvedilol patients compared with two propranolol patients because of new-onset or worsening ascites or ankle edema. The new onset/worsening ascites have been attributed to a possible increase in sodium retention [29] and the need for observation as the new onset/worsening ascites take weeks to present, as it was not noted in the first week [28]. However, it is worth noting that there was no difference in the new onset or worsening of ascites between the study groups in a randomized clinical trial investigating the use of carvedilol in primary and secondary prevention of variceal bleeding. The carvedilol dose in both studies was 12.5 mg/day, which suggests that the worsening of sodium and water retention may not be due to carvedilol because of the relatively low carvedilol doses [10]. In the study by Sharma et al., it was found that there were fewer ascites in patients taking carvedilol, and new or worsening ascites were more common in those receiving propranolol (69.5% vs. 40%; p = 0.04). Other clinical decompensations and complications of liver disease occurred at comparable rates between the two groups. Drug-related adverse events were similar in both groups [32].

Limitations

This study is limited because the acquired results were from single-center studies with small study populations and varying follow-up courses. Notably, there is significant heterogeneity in the results reported between the studies due to differences in the dose and frequency of carvedilol and propranolol.

## Conclusions

Liver cirrhosis is the most common cause of portal hypertension. Portal hypertension has devastating consequences in cirrhotic patients, such as the development of varices and subsequent variceal bleeding. Portal hypertension is measured through the hepatic venous pressure gradient. Pharmacological management with nonselective β-adrenergic blockers is used in cirrhotic patients to lower portal hypertension and as primary and secondary prophylaxis for variceal bleeding. Propranolol was the oldest and most commonly used nonselective β-adrenergic blocker to lower portal hypertension and reduce the risk of variceal bleeding. Clinical trials have shown that carvedilol is more effective than propranolol in reducing HVPG and the risk of variceal bleeding. Given the clinical trial results, carvedilol should be considered an effective alternative to propranolol in cirrhotic patients with portal hypertension and varices. However, large-scale multicenter studies with extended follow-up periods are warranted to assess this thoroughly.
